# Supplementary material for: Unexpected Crosslinking Effects of a Human Thyroid Stimulating Monoclonal Autoantibody, M22, with IGF1 on Adipogenesis in 3T3L-1 Cells
Source: Int J Mol Sci. 2023 Jan 6;24(2):1110. doi: 10.3390/ijms24021110 (PMC9863235; doi:10.3390/ijms24021110)
Supplement: Supplementary file 1 [file ijms-24-01110-s001.zip › ijms-2096274-supplementary.pdf]

Supplemental materials

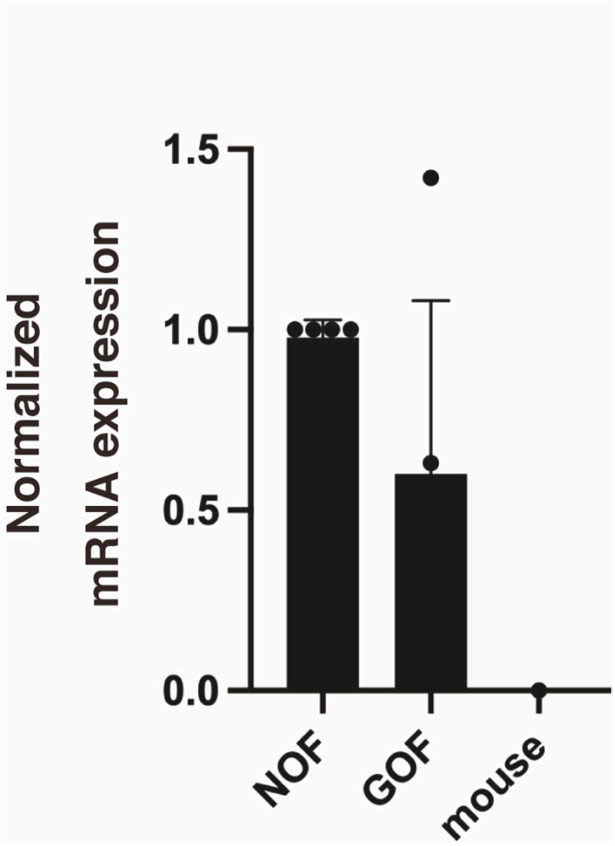

Supplemental Figure S1. mRNA expression of THSR by 2D cultured 3T3-L1 cells, non-GO-related human orbital fibroblasts (n-HOFs) or GO-related HOFs (GOHFs).

2D cultured 3T3-L1 cells, non-GO-related human orbital fibroblasts (n-HOFs) or GO-related HOFs (GOHFs) were subjected to qPCR analysis and the expression of the mRNA for mouse *TSHR or* human *THSR* was estimated for 3T3-L1 cells or n-HOFs and GHOFs, respectively. All experiments were performed in duplicate using 3 different confluent 6-well dishes (2D) in each experimental condition. *P<0.05.

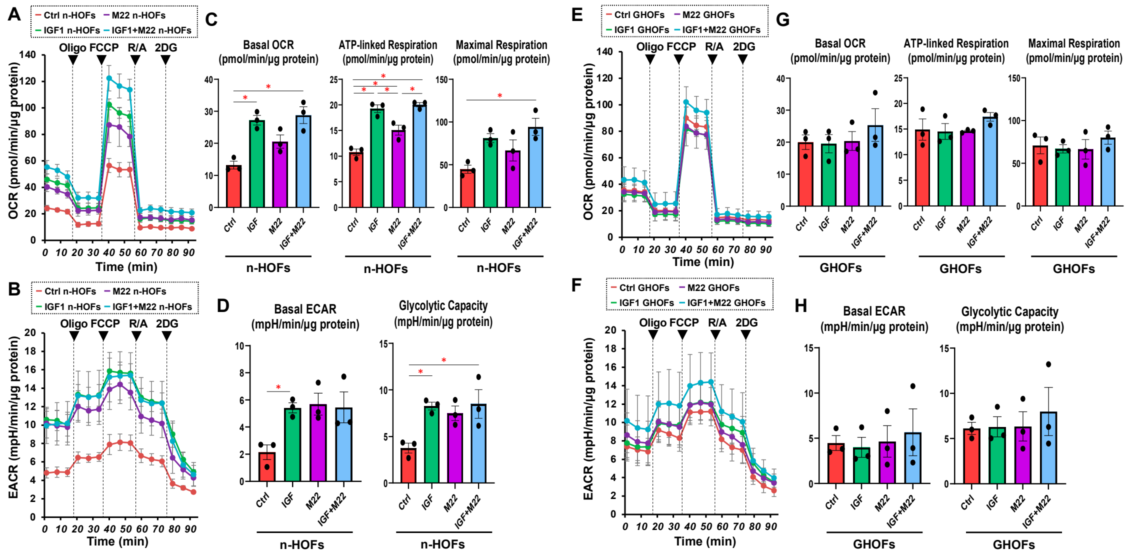

Supplementary Figure S2. Effects of IGF1 and/or M22 on cellular metabolism in non-GO-related human orbital fibroblasts (n-HOFs) and GO-related HOFs (GOHFs).

In the presence or absence of 100 µg/ ml IGF1 and/or 10 µg ml M22, the 2D cultured human orbital fibroblasts (HOFs) were subjected to a real-time metabolic function analysis using a Seahorse XFe96 Bioanalyzer. Results for non-GO HOFs (n-HOFs) are shown in panels A-D, and results for GO-related HOFs (GOHFs) are shown in panels E-H. The rates of oxygen consumption rate (OCR, panel A and E) and extracellular acidification (ECAR, panel B and F) were measured, and thereafter they were further measured after subsequent supplementation with oligomycin (complex V inhibitor), FCCP (a protonphore), and rotenone/antimycin A (complex I/III inhibitors) and 2DG (hexokinase inhibitor). Key parameters of mitochondrial respiration are shown in panels C and panel G, and those of glycolytic flux are shown in panel D and H, respectively. Basal OCR was calculated by subtracting OCR with rotenone/antimycin A from OCR at baseline. ATP-linked respiration was calculated by subtracting OCR with oligomycin from OCR at baseline. Maximal respiration was calculated by subtracting

OCR with rotenone/antimycin A from OCR with FCCP. Basal ECAR was calculated by subtracting ECAR with 2DG from ECAR at baseline. Glycolytic capacity was calculated by subtracting ECAR with 2DG from ECAR with oligomycin. Glycolytic reserve was calculated by subtracting ECAR at baseline from ECAR with oligomycin. All experiments were performed in triplicated using fresh preparations (n=3). *P<0.05. Ctrl = the absence of IGF1 and M22.

Supplementary Methods

<u>Adipocyte Culture and Adipogenic Differentiation</u>

The 3T3-L1 cell (#EC86052701-G0, KAK) is a cell line that is universally used in the field of lipid research. The 3T3-L1 preadipocytes were grown until confluence at 37°C in HG- DMEM containing 8 mg/L d-biotin, 4 mg/L calcium pantothenate, 100 U/mL penicillin, 100 µg/mL streptomycin (b.p. HG-DMEM), and 10% CS.

The 3T3-L1 organoids were generated using a hanging droplet spheroid three-dimension (3D) culture system as described in a recent report. [29] Briefly, to facilitate stable morphology, methylcellulose (Methocel A4M) was added to the growth medium. Prior to seeding the hanging drop culture plate (# HDP1385, Sigma-Aldrich), cells were cultured in 100 mm or 150 mm dishes until reaching approximately 90% confluence. After washing with phosphate buffered saline (PBS), the cells were detached using 0.25% Trypsin/EDTA and resuspended in growth medium. After centrifugation for 5 min at 300 g, the cell pellet was re-suspended in growth medium containing 0.25% w/v Methocel A4M. The volume was adjusted so that 20,000 cells were contained in 28µL of the solution, and 28 µL drops were placed into each well of the drop culture plate (defined as 3D/Day 0). An organoid medium (i.e. growth medium with 0.25% w/v Methocel A4M)

was used throughout the duration of the spheroid culture. On each following day, 14 μL of the culture medium was removed and a fresh 14 μL of culture medium was added to each well.

Isolation of GHOFs was performed by a previously described method using surgically obtained orbital fat explants from 4 patients with GO [29, 31]. Isolation of HOFs was performed by a previously described method using surgically obtained orbital fat explants from 4 non-GO patients with orbital fat herniation. Thereafter, their 2D planer culture was basically processed as described above.

Seahorse real time cellular analysis

Approximately, 20,000 2D-cultured 3T3-L1 cells and HOFs were placed in the wells of a 96-well assay plate. After replacing the culture medium with Seahorse XF DMEM assay medium (pH 7.4, Agilent Technologies, #103575-100) supplemented with 5.5 mM glucose, 2.0 mM glutamine, and 1.0 mM sodium pyruvate, the basal OCR and ECAR values were determined using a Seahorse XFe96 Bioanalyzer and the samples were then further analyzed after supplementation with 2.0 μM oligomycin, 5.0 μM carbonyl cyanide p-trifluoromethoxyphenylhydrazone (FCCP), 1.0 μM rotenone and antimycin A, and 10mM 2-deoxyglucose (2-DG). The OCR and ECAR values were normalized to the amount of protein per well, as assessed by BCA protein assay (TaKaRa Bio Inc., Shiga, Japan) after completion of the assay.

Lipid staining

Lipid staining of the 2D cultured 3T3-L1 cells by an Oil Red O staining assay was performed using a commercial kit (Abcam, #133102). Microscopy images were

obtained by a Nikon A1 confocal microscope (Tokyo, Japan) and quantification was performed by measuring optical density (O.D.) of the dissolved dye at 500 nm.

Alternatively, the lipid staining of the 4 % paraformaldehyde (PFA) fixed 3D spheroids were processed in a mixture of 0.1 % BODIPY (# D3922, Thermo Fisher Scientific), 0.1 % DAPI (#D523, Doujin, Japan) and 0.1 % phalloidin (#20553, Funakoshi, Japan) in phosphate buffered saline (PBS) containing 3 % bovine serum albumin (BSA) for 3 hrs. The fluorescence intensity was measured using a Nikon A1 confocal microscope (Tokyo, Japan) was quantified using the Image J software version 2.0.0 (NIH, Bethesda, MD).

Immunocytechemistry

The 2D and 3D 3T3-L1 cells were immune-stained using a primary antibody (1 : 200 dilutions); a rabbit anti-collagen monoclonal antibody (collagen 1 ; # 600-401-103-0.1 or collagen 4; # 600-401-106-0.1, Rockland Immuno-chemicals Inc.) and the 2nd antibody (1 : 500 dilutions) of goat Alexa Fluor 488 anti-rabbit IgG (# A-11070, Thermo Fischer Scientific) or goat Alexa Fluor 594 anti-mouse IgG (# A-11020, Thermo Fischer Scientific) with Alexa Fluor 594 phalloidin (# 20553, Funakoshi) and DAPI (# D523, Dojindo) at 1:1000 dilutions for 3 hrs at room temperature as described previously [24]. The fluorescence intensity of each ECM labeling was determined using a Nikon A1 confocal microscope (Tokyo, Japan) and quantified using the Image J software version 2.0.0 (NIH, Bethesda, MD).

Quantitative PCR

Using total RNA extraction by a RNeasy mini kit (Qiagen, Valencia, CA) and

reverse transcription by a SuperScript IV kit (Invitrogen), the samples were processed according to the manufacturer's instructions. The real-time PCR with the Universal Taqman Master mix using a StepOnePlus instrument (Applied Biosystems/Thermo Fisher Scientific) was performed. cDNA levels, expressed as fold-change relative to the expression of a housekeeping 36B4 (*Rplp0*) gene, were then calculated. Sequences of the primers and Taqman probes used are shown in Supplementary Table 1.

Micro-indentation force measurement

The micro-indentation force of the spheroid was measured using a micro-squeezer (CellScale, Waterloo, ON, Canada) as described previously [29]. Briefly, a single spheroid was placed on a 3-mm × 3-mm plate and then compressed to a 50 % deformation during a period of 20 seconds under monitoring by a micro-camera. The required strain (μN) was measured, and force/displacement (μN/μm) was calculated.

Statistical analysis

All statistical analyses were performed using Graph Pad Prism 8 (GraphPad Software, San Diego, CA). To analyze the difference between groups, a grouped analysis with two-way analysis of variance (ANOVA) followed by a Tukey's multiple comparison test was performed. Data are presented as the arithmetic mean ± the standard error of the mean (SEM).

Supplemental Table S1

Supplemental Table S1. Primers for qPCR.

Gene	Forward primer (5' to 3')	Reverse primer (5' to 3')	TaqMan probe (5' to 3')
**Mouse**			
36b4	TTATAACCCTGAAGTG CTCGAC	CGCTTGTACCCATT-GATGATG	/FAM/AG-GCCCTGC/ZEN/ACTCTCGCTT/IABkFQ/
Col1a1	CGCAAAGAGTC-TACATGTCTAGG	CATTGTG-TATGCAGCTGACTTC	/FAM/CCGGAGGTC/ZEN/CACAAA-GCTGAACA/IABkFQ/
Col4a1	TCTGGCTGTG-GAAAATGTGA	AATCCAATGACAC-CTTGCAAC	/FAM/TCTTTCTCC/ZEN/CTTTT-GTCCCTTCACGC/IABkFQ/
Col6a1	CCAGATGAGTGTGA-GATCCTG	AAGTTCTG-TAGGCCAATGCTC	/FAM/ACCCATTGA/ZEN/CATCCTCTTCGT GCTG/IABkFQ/
Fn1	GAGCTATCCATTTCAC-CTTCAGA	TTGTTCGTAGACAC-TGGAGA	/FAM/CAGGAGATT/ZEN/TGTTAGGAC-CACGGCA/IABkFQ/
Pparg	CTGCTCCACAC-TATGAAGACAT	TGCAGGTTCTACTTT-GATCGC	/FAM/AGCTGACCC/ZEN/AATGGTT-GCTGATTACA/IABkFQ/
Fabp4	AAATCACCGCAGAC-GACAG	CCTTTCATAACACATT CCACCAC	/FAM/TGAAGAGCA/ZEN/TCATAACCCTA-GATGGCG/IABkFQ/
